# Incidence rates of in-hospital carpal tunnel syndrome in the general population and possible associations with marital status

**DOI:** 10.1186/1471-2458-8-374

**Published:** 2008-10-28

**Authors:** Stefano Mattioli, Alberto Baldasseroni, Stefania Curti, Robin MT Cooke, Antonella Bena, Giovanna de Giacomi, Marco dell'Omo, Pirous Fateh-Moghadam, Carla Melani, Marco Biocca, Eva Buiatti, Giuseppe Campo, Francesca Zanardi, Francesco S Violante

**Affiliations:** 1 Occupational Medicine Unit, Dipartimento di Medicina Interna, dell'Invecchiamento e Malattie Nefrologiche, University of Bologna, Italy; 2 Tuscany Regional Centre for Occupational Injuries and Diseases (CeRIMP), Florence, Italy; 3 Occupational Epidemiology Unit, Piedmont Region, Grugliasco, Turin, Italy; 4 National Agency for Regional Health Services, Rome, Italy (during data collection, Marche Regional Health Care Agency, Ancona, Italy); 5 Institute of Occupational Medicine and Toxicology, University of Perugia, Perugia, Italy; 6 Epidemiology Unit, Azienda Provinciale per i Servizi Sanitari, Provincia Autonoma di Trento, Italy; 7 Epidemiology Unit, Assessorato alla Sanità e Politiche Sociali, Provincia Autonoma di Bolzano, Italy; 8 Emilia Romagna Regional Health Care Agency, Bologna, Italy; 9 Tuscany Regional Health Care Agency, Florence, Italy; 10 Dipartimento Processi Organizzativi, National Institute of Occupational Safety and Prevention (ISPESL), Rome, Italy; 11 Stefano Mattioli, Unità Operativa di Medicina del Lavoro, Policlinico Sant'Orsola-Malpighi, Dipartimento di Medicina Interna, dell'Invecchiamento e Malattie Nefrologiche, Università di Bologna, via Pelagio Palagi 9, I-40138 Bologna, Italy

## Abstract

**Background:**

Carpal tunnel syndrome (CTS) is a socially relevant condition associated with biomechanical risk factors. We evaluated age-sex-specific incidence rates of in-hospital cases of CTS in central/northern Italy and explored relations with marital status.

**Methods:**

Seven regions were considered (overall population, 14.9 million) over 3–6-year periods between 1997 and 2002 (when out-of-hospital CTS surgery was extremely rare). Incidence rates of in-hospital cases of CTS were estimated based on 1) codified demographic, diagnostic and intervention data in obligatory discharge records from all Italian public/private hospitals, archived (according to residence) on regional databases; 2) demographic general population data for each region. We compared (using the χ_score _test) age-sex-specific rates between married, unmarried, divorced and widowed subsets of the general population. We calculated standardized incidence ratios (SIRs) for married/unmarried men and women.

**Results:**

Age-standardized incidence rates (per 100,000 person-years) of in-hospital cases of CTS were 166 in women and 44 in men (106 overall). Married subjects of both sexes showed higher age-specific rates with respect to unmarried men/women. SIRs were calculated comparing married vs unmarried rates of both sexes: 1.59 (95% confidence interval [95% CI], 1.57–1.60) in women, and 1.42 (95% CI, 1.40–1.45) in men. As compared with married women/men, widows/widowers both showed 2–3-fold higher incidence peaks during the fourth decade of life (beyond 50 years of age, widowed subjects showed similar trends to unmarried counterparts).

**Conclusion:**

This large population-based study illustrates distinct age-related trends in men and women, and also raises the question whether marital status could be associated with CTS in the general population.

## Background

Carpal tunnel syndrome (CTS) is a socially relevant work-related disabling condition [1,2], with biomechanical overload being a major risk factor [3]. The social costs of CTS include lost working days, changes of occupation and frequent need for surgical treatment [4]. CTS affects women more than men, with a peak incidence occurring at peri-menopausal age (in contrast to a gradually increasing age-related trend in men) [5-7]. The reported overall prevalence of clinically/instrumentally diagnosed CTS among the general population in southern Sweden was 2.7% [8]. Incidence of clinically/instrumentally diagnosed cases of CTS in the general population of Siena (Italy) was 329/100,000 person-years (with women 3.6-fold more affected than men) [5]. Regarding surgically treated CTS, a statewide incidence of 144 per 100,000 inhabitants was reported for 1993 in the general population of Maine [9].

We evaluated age-sex-specific incidence rates of in-hospital cases of CTS in seven administrative regions of central/northern Italy. Based on data availability considerations and our interest in the possible role of household chores as a biomechanically plausible risk factor for CTS [10], we also decided to stratify incidence rates by marital status.

## Methods

### Setting and Survey

**Figure 1:**
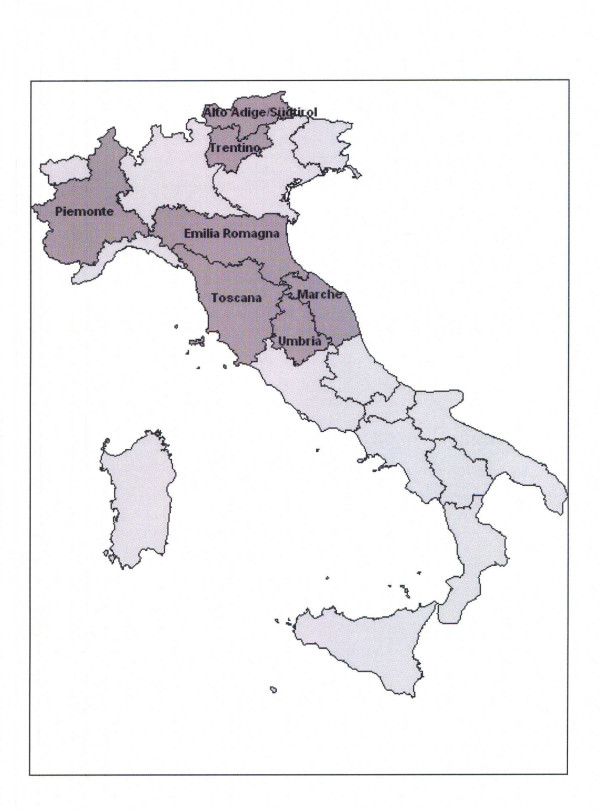
The seven regions included in the study (dark shading): Piemonte (4.2 million inhabitants); Emilia-Romagna (4.0 million); Toscana (3.5 million); Marche (1.5 million); Umbria (0.8 million); Alto-Adige/Südtirol (0.5 million); Trentino (0.5 million).

In Italy, both public and private hospitals obligatorily provide individual discharge records–even for surgically treated day patients–containing codified demographic information (including age, gender, address of primary residence, and marital status) transcribed from identity cards that all residents are obliged to obtain from their local Municipalities, who in turn collate and regularly communicate their anagraphic data to The National Institute of Statistics (ISTAT). The information on each hospital discharge record is registered in databases of the patients' region of residence, irrespective of hospital location. During the periods under consideration, carpal tunnel release operations in Italy were almost invariably conducted on public/private hospital premises after severe chronic symptoms and positive nerve conduction studies. We reviewed the records of all patients with a principal diagnosis of CTS (ICD-9 code 354.0) in seven administrative areas (Figure 1): Piemonte (in 1997–2001), Emilia-Romagna (1997–2000), Toscana (1997–2000), Marche (1997–2002), Umbria (1999–2001), Alto-Adige/Südtirol (1998–2002) and Trentino (1997–2002). The overall population of the study area was 14.9 million inhabitants (at the 2001 population census) [11]; thus almost 68 million person-years were considered. Repeated admissions were excluded. For all regions except Umbria, self-reported *de jure *marital status at the time of admission was available in terms of 'unmarried' (including cohabitant subjects), 'married' (including separated partners), 'widowed', or 'divorced' (divorced status was considered only for younger age-groups, following the 1970 Italian divorce law). Demographic general population data in each region (for each relevant year) were obtained from ISTAT (based on the demographic data regularly provided and updated by all Italian Municipalities) [11].

### Statistical Analysis

We calculated age-sex-specific rates and standardized rates (age-adjusted by the Standard European Population proposed by the WHO) [12]. Age-sex-specific rates with respect to marital status were calculated, and the χ_score _test [13] was used to evaluate differences. An overall comparison between unmarried and married rates was obtained using indirect standardization [12]: standardized incidence ratios (SIRs) were calculated as comparable measures, using age-sex-specific rates among the unmarried as standard rates. To compare in-hospital and clinically/electrodiagnostically diagnosed CTS rates, we examined hospital discharge records of the area of Siena (Local Health District, *Zona Senese*) considered in a previous study [5] in corresponding years (1997–1998) and compared annual crude and sex-specific rates. Stata 9.0 SE (Stata Corporation, Texas, TX) was used for all statistical analysis, with significance set at P < 0.05.

## Results

### Survey

Excluding repeated admissions, 86,641 in-hospital cases of CTS were identified, 79% of whom (n = 68,361) were women. At least 96% (n = 82,743) of the patients received specific surgical treatment (Diagnosis Related Group [DRG] code 006, "Carpal Tunnel Release").

### Incidence of CTS

**Figure 2:**
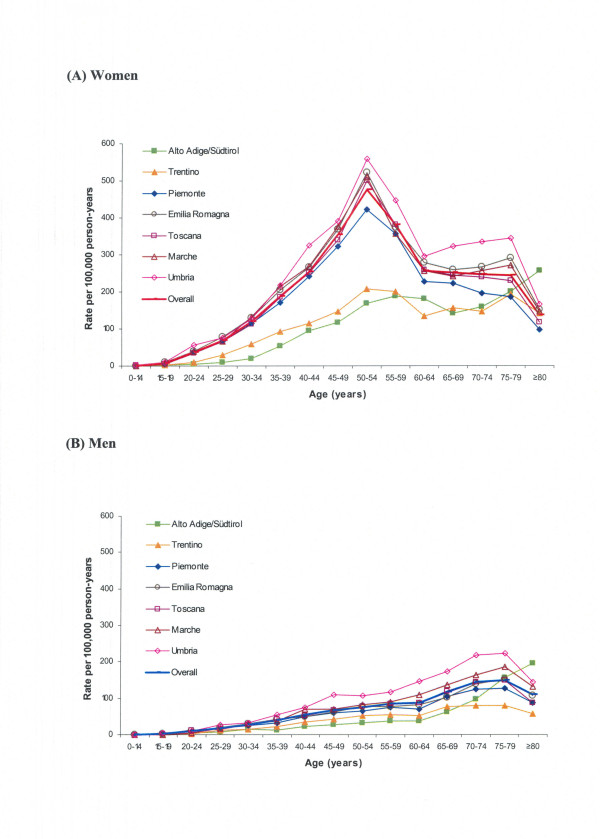
Age-specific rates of in-hospital cases of CTS in different regions in (A) women and (B) men.

The overall age-standardized incidence rate of in-hospital cases of CTS was 106.09 per 100,000 person-years (95% confidence interval [95% CI], 106.09-106.09), ranging from 49.64 (95% CI, 49.62–49.66) in Alto-Adige/Südtirol to 132.47 (95% CI, 132.45–132.49) in Umbria. The age-standardized incidence rate for women was 3.8-fold that for men: 166.27 per 100,000 person-years (95% CI, 166.27–166.28) vs 44.11 (95% CI, 44.10–44.11). Analogous sex-related differences were recorded in each region (data not shown). Figure 2 reports age-sex-specific rates in the different regions and for the overall study population. Among women, marked incidence peaks were observable in the 50–54-year age group in all regions except Trentino and Alto-Adige/Südtirol (which showed less pronounced peaks around 50–59 years). A different pattern was observable for men, who showed an increasing trend until age 75–79 years.

### Differences in Marital Status

**Figure 3:**
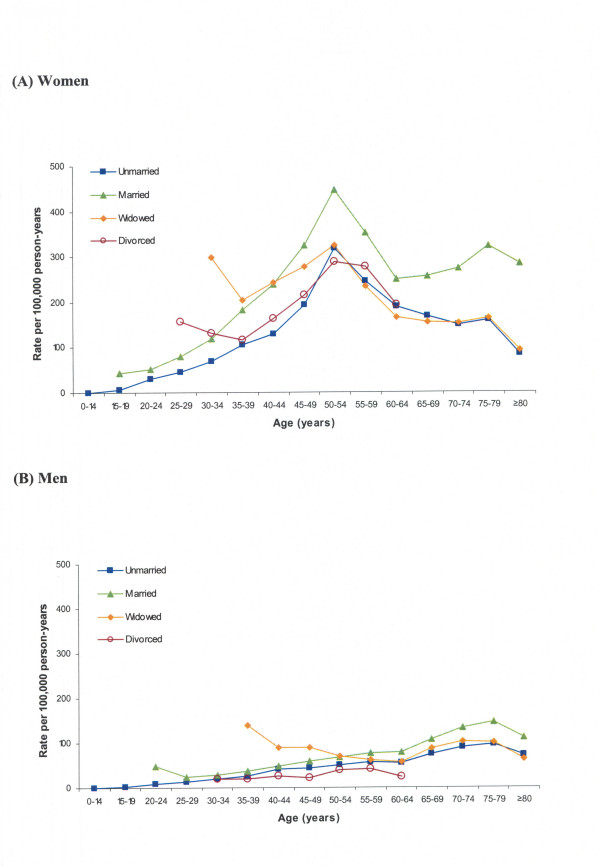
**Age-specific rates of in-hospital cases of CTS according to marital status in (A) women and (B) men.** (For divorced subjects, only younger age-groups–emerging after the introduction of the 1970 Italian divorce law–were analyzed.)

Figure 3 shows age-specific rates in the overall study population according to marital status. As compared with their unmarried counterparts, spouses of both sexes showed significantly higher age-specific rates (Tables 1 and 2). SIRs for married vs unmarried rates were 1.59 (95% CI, 1.57–1.60) in women and 1.42 (95% CI, 1.40–1.45) in men. Young widows and widowers showed higher incidence peaks in comparison with their married counterparts (2–3-fold higher rates at the age of 30–34 years [women] or 35–39 years [men], albeit with wide 95% CI). Remarkably, widowed men/women aged over 50 years showed similar trends to unmarried men/women. With regard to divorced status, very young divorced women (aged 25–34-years) showed 2- to 3-fold higher rates (though with wide 95% CI) than their unmarried counterparts, whereas divorced women aged over 35 years showed similar trends to unmarried women; divorced men showed lower trends than unmarried men.

**Table 1:** Age-specific rates per 100,000 person-years (with 95% CI) of in-hospital cases of CTS according to marital status among women

*Age (years)*	*Unmarried*	*Married*	*Widowed*	*Divorced*
0–14	0.9 (0.7–1.3)	-	-	-
15–19	6 (4–7)	43 (16–115)^a^	-	-
20–24	29 (27–32)	52 (43–63)^a^	-	-
25–29	45 (42–49)	80 (75–86)^a^	-	157 (87–284)^c, d^
30–34	68 (62–74)	118 (114–124)^a^	297 (208–425)^b^	130 (97–174)^d^
35–39	106 (97–117)	182 (176–188)^a^	203 (151–274)	116 (94–144)^c^
40–44	130 (116–145)	238 (231–245)^a^	242 (198–297)	164 (139–193)^c, d^
45–49	194 (174–217)	323 (315–332)^a^	277 (241–319)^b^	215 (186–249)^c^
50–54	317 (289–348)	447 (438–457)^a^	324 (294–356)^b^	288 (252–329)^c^
55–59	245 (219–274)	351 (342–360)^a^	232 (215–255)^b^	277 (236–326)^c^
60–64	188 (167–213)	248 (241–256)^a^	165 (153–178)^b^	192 (154–240)^c^
65–69	167 (148–189)	254 (245–263)^a^	155 (146–166)^b^	-
70–74	148 (130–166)	273 (263–283)^a^	152 (144–161)^b^	-
75–79	159 (141–179)	322 (308–337)^a^	163 (155–171)^b^	-
≥ 80	84 (74–97)	283 (264–302)^a^	92 (87–97)^b^	-

Significant difference at χ_score _test: ^a^married vs unmarried; ^b^widowed vs married; ^c^divorced vs married; ^d^divorced vs unmarried. Empty cells are due to lack of data, unreliable data (elderly divorcees) or restricted numbers (young widows).

**Table 2:** Age-specific rates per 100,000 person-years (with 95% CI) of in-hospital cases of CTS according to marital status among men

*Age (years)*	*Unmarried*	*Married*	*Widowed*	*Divorced*
0–14	0.4 (0.2–0.6)	-	-	-
15–19	2 (1–3)	-	-	-
20–24	8 (7–9)	47 (32–71)^a^	-	-
25–29	13 (12–15)	25 (21–29)^a^	-	-
30–34	21 (18–23)	29 (26–32)^a^	-	21 (7–64)
35–39	26 (23–30)	37 (34–40)^a^	138 (62–308)^b^	19 (10–38)^c^
40–44	40 (35–47)	49 (46–52)^a^	89 (42–186)	26 (16–43)^c^
45–49	44 (37–52)	59 (56–63)^a^	89 (52–154)	21 (12–35)^c, d^
50–54	50 (42–60)	68 (65–72)^a^	69 (44–108)	38 (26–57)^c^
55–59	56 (46–68)	76 (72–80)^a^	60 (41–88)	41 (26–65)^c^
60–64	54 (45–66)	79 (75–83)^a^	56 (41–77)^b^	24 (12–49)^c, d^
65–69	74 (62–89)	107 (102–112)^a^	87 (71–107)	-
70–74	88 (74–106)	133 (127–140)^a^	103 (87–121)^b^	-
75–79	95 (76–118)	146 (138–154)^a^	100 (85–117)^b^	-
≥ 80	72 (55–94)	111 (104–120)^a^	64 (56–73)^b^	-

Significant difference at χ_score _test: ^a^married vs unmarried; ^b^widowed vs married; ^c^divorced vs married; ^d^divorced vs unmarried. Empty cells are due to lack of data, unreliable data (elderly divorced men) or restricted numbers (young married men).

### In-Hospital vs Diagnostic Rates of CTS

Comparison of clinically/electrodiagnostically diagnosed CTS rates in Siena [5] with the in-hospital rates recorded by us in the same zone (and years) suggested a 2 to 3-fold difference. Overall crude incidence rates (per 100,000 person-years) of in-hospital CTS in the zone were 160 for 1997 and 129 for 1998, as compared with 327 and 345, respectively, for clinically/electrodiagnostically diagnosed CTS [5].

## Discussion

This large population-based study of rates of in-hospital CTS in central/northern Italy reinforces our knowledge of age- and sex-related trends, and suggests that marital status might be associated with clinically/socially relevant chronic CTS.

**Figure 4:**
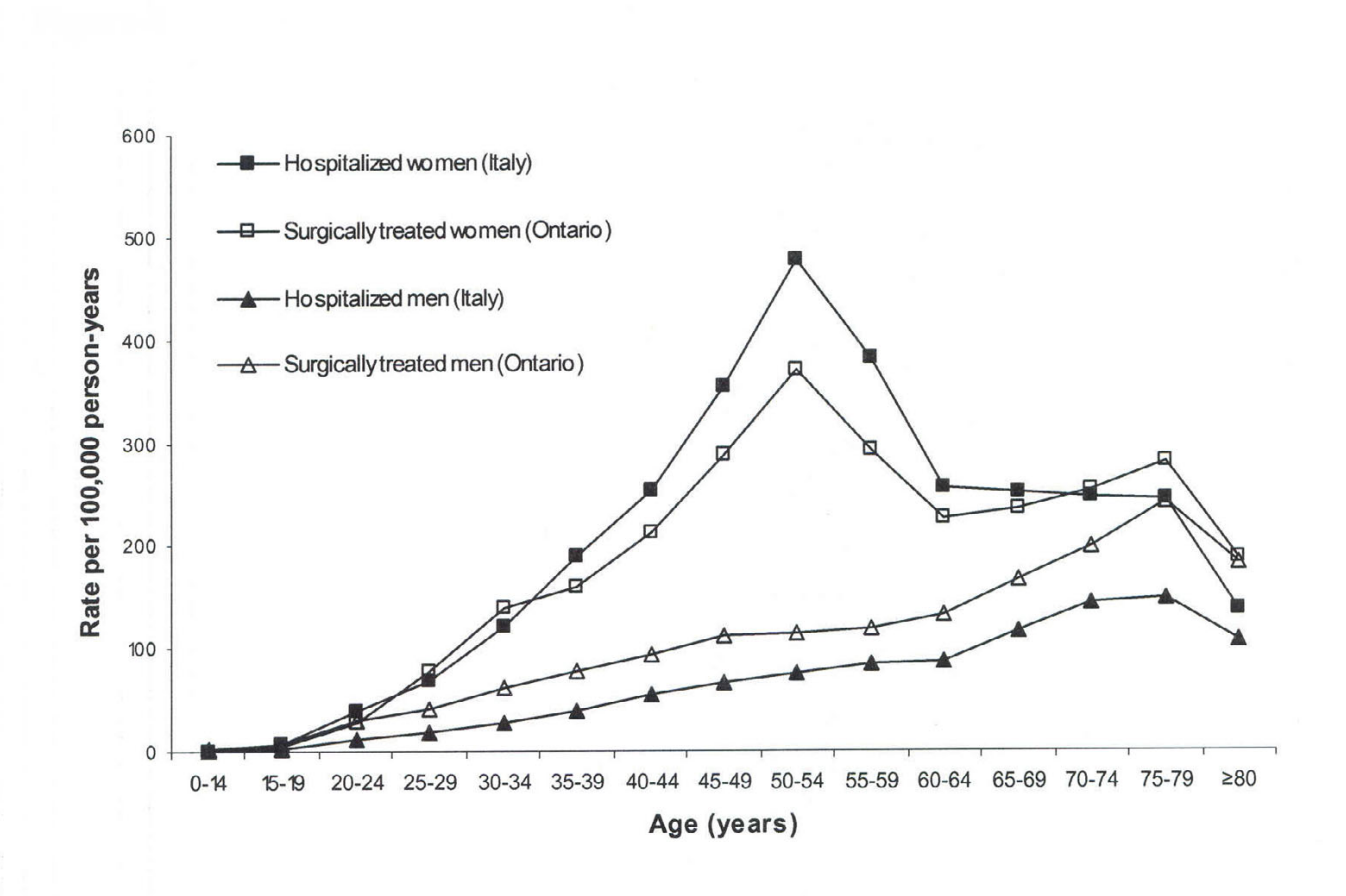
**Age-sex-specific rates of in-hospital CTS in the present study, as compared with rates reported for Ontario in 1988**[7].

The overall age-standardized incidence rate of 106 in-hospital cases of CTS per 100,000 person-years is lower than the rate of 144 surgically treated cases per 100,000 reported for the state of Maine in 1993 [9] (when according to 1990/2000 census data the state population was 1.2–1.3 million inhabitants) [14]. The rate for Maine is rather similar to that of Umbria, but more than 2-fold higher than that of Alto-Adige/Südtirol. Such regional variations (reported also for small areas within Maine) can likely be attributed to socio-economic, occupational, environmental and health-care differentials [9], including access to care, diagnosis, and practice patterns (attitudes towards advising more conservative approaches, etc.) [6,15,16]. Age-related trends (Figure 2) were remarkably similar in most of the regions studied. In line with other reports [5-7], men displayed gradually increasing incidence until advanced age, whereas women showed a sharp perimenopausal peak (corresponding to the 50–54 year age group) after progressively increasing incidence during the fertile years. These observations are broadly consistent with the concept that in women there may be a hormonal component in the etiology of CTS, perhaps involving long-term hormonal effects of pregnancy or cumulative exposure to female sex hormones [17,18]. Remarkably, comparison with age-sex-specific rates of surgically treated CTS in Ontario [7] reveals almost superimposable trends (Figure 4).

Analysis of data regarding Siena [5] suggests a proportion of conservative treatment of 50% or more, as far as it is possible to estimate from a comparison of two different studies. This figure is broadly in line with a comparison of neurophysiologically confirmed and surgically treated CTS rates in East Kent (England) [6], but somewhat higher than the proportion (31%) of surgically treated cases of CTS reported in a recent study of incidence compressive neuropathies in UK general practices [19]. It should also be remembered that the prevalence of symptomatic cases of CTS in the general population appears to be considerably higher than that of diagnosed cases [8].

As regards marital status, married women and men in all age groups turned out to have higher SIRs as compared with their unmarried counterparts (with overall excesses of 60% for women and 40% for men). Since married status has been associated with favourable levels of general health [20] (apart from cancer [21]) and better socioeconomic and occupational conditions [22], these findings could be considered unexpected. However, marital status could be a marker of several relevant risk factors for CTS, including parity, high body mass index [23] and at-risk occupations. In particular, part of the excess incidence of in-hospital CTS among married women could be attributed to higher parity in the years after marriage (of note, in a case-control study of CTS in industrial workers, parity ≥ 3 turned out to be a relevant risk factor) [24]. The higher rates found in married women and men of different ages in comparison with their unmarried/divorced counterparts might also be partially related to higher body mass index among married couples. Whereas in the U.S.A. a cross-sectional analysis of National Health Interview Surveys' data (1999–2002) according to marital status did not find a significant excess of overweight/obese married women [22], in Spain a greater prevalence of obesity has been reported among married men and women up to the age of 45 years [25] (we were unable to find any analogous information for Italy). On the other hand, the remarkably high incidence recorded for married men in their twenties could be related to having to work (presumably often manually, given their relatively young age) to support a family.

A possible role of housekeeping chores [10] may also deserve some consideration. However, housework is generally less strenuous and repetitive than industrial work and, to our knowledge, only an isolated case-control study among Beijing women [26] suggested any association between manual household tasks and CTS. Nevertheless, we think that manual domestic chores might conceivably be of some relevance in two circumstances: 1) among manual workers who experience an additional biomechanical exposure on top of their professional exposure; 2) among house-proud full-time housewives who have concurrent risk factors for CTS. However, it would be difficult to explain the excess incidence of in-hospital CTS among married men in these terms (even though men can help around the house in a variety of ways, including maintenance, manual handling, etc.).

Another possibility is that homemaking-related factors might affect rates of in-hospital CTS through increased obligations: the need to remain able to perform essential household chores might provide an incentive for married women/men with homemaking responsibilities and young divorced/widowed people to seek surgical treatment. Conversely, unmarried people might be better placed to limit household activities in order to postpone or avoid surgical treatment. Interestingly, we recorded high in-hospital CTS rates among young divorced women, widows and widowers, who are likely to assume increased responsibility for the running of households with children. The absence of similar trends among divorced men, older widows/widowers, and divorced women aged >50 years (who showed incidence rates very similar to those of unmarried subjects of the same sex and age) are also intriguing. It will be interesting to see whether some or any of these trends are reproduced in different national settings.

### Study limitations

The ecological study design precluded analysis at an individual level. Elevated rates of CTS might also be attributed to general hospitalization trends related to marital status. We therefore examined the hospital discharge records of all patients hospitalized in Emilia-Romagna during the study period (excluding repeated admissions) with any principal diagnosis except delivery, complications of pregnancy or abortion (data not shown). Apart from the elderly (≥ 65 years) age group (where, in line with a previous report [27], married status was associated with higher rates of hospitalization), patterns of hospital admission were broadly similar for married and unmarried subjects, with a slight significant excess among unmarried inpatients between the age of 45 and 65 years. It could be argued that the consistent pattern between married/unmarried patients across age groups points might reflect a systematic bias derived from self-reporting of *de jure *marital status at the time of hospital admission. However, we are unable to think of any motive for inexact self-reporting (except perhaps concealment of divorced status among some particularly religiously observant patients, which would have led to underestimate of risk among divorced subjects).

The restricted information contained in the hospital discharge records impeded analysis of potential interactions or confounding with occupational and lifestyle factors. A subanalysis of self-reported 'main professional role' (feasible only for Tuscany) showed only minor differences in distribution of white-collar workers versus blue-collar workers or housewives in the 'married' and 'unmarried' subsets of patients, which would not be sufficient to explain the differences in hospitalization rates (data not shown).

Although the compulsory hospital discharge records are institutionally standardized, their reliability could be compromised by errors or omissions (in other respects, we do not think missing data should be a major concern). However, there was a 96% concordance rate between the disease identification code (ICD-9) and the DRG code for carpal tunnel release (and other recorded DRG codes also appeared appropriate). Since ISTAT does not provide information regarding 'separated' or 'cohabitant' marital status, it was not possible to take these factors into account in the analysis (thus, 'separated' individuals were likely to be included in the 'married' category, and 'cohabitant' individuals mainly in the 'unmarried' subset). Furthermore, the registration of patients' marital status only at the time of admission to hospital (without historical reconstruction of status changes and durations in each condition) could have led to a non-differential misclassification of exposure among patients who had recently changed status. These factors may have led to underestimates in the observed differences between rates associated with 'married' and 'unmarried' status. Due to number limitations, some of the age-sex-specific estimates for marital status showed wide 95% CI (reported in Tables 1 and 2) and caution is needed when interpreting comparisons.

It is also important to underline that this study regards rates of hospital admissions due to CTS (in the vast majority of cases for the purposes of treatment) rather than incidence of all clinically relevant cases of CTS. Knowledge of Italian practice during the period suggests that the hospital discharge records mainly correspond to highly symptomatic patients who eventually elected to undergo surgical treatment after several years of discomfort and positive nerve conduction study findings [28]. This observation is broadly in line with concepts expressed elsewhere [29,30]. Therefore, it is reasonable to suppose that the reported rates may regard severely symptomatic, socially relevant chronic CTS [30].

## Conclusion

In summary, this large population-based study provides important confirmation of distinct age-related trends in men and women. Our findings also raise the question as to whether marital status could be associated with CTS in the general population. Studies in other national settings could explore this possibility. In the meantime, it should be underlined that our findings regarding marital status must be considered preliminary and merely hypothesis generating, especially given the absence in the present work of data regarding individual/occupational factors (or biomechanical exposures) and the lack of information about those cases of CTS which do not reach hospital treatment.

## Competing interests

The authors declare that they have no competing interests.

## Authors' contributions

SM, ABa and GC designed the study with SC, ABe, GdG, MdO, PFM, CM and MB.

SM, ABa, ABe, GdG, MdO, PFM and CM were responsible for supervising data collection in the different centers. SM, SC and GC were responsible for data analysis. SM, SC and RMTC drafted the manuscript and contributed to interpretation, together with ABa, MB, EB, FZ and FSV. FSV supervised the entire work. All authors critically revised the manuscript. All authors read and approved the final manuscript.

## Pre-publication history

The pre-publication history for this paper can be accessed here:


